# The role of Hedgehog and Notch signaling pathway in cancer

**DOI:** 10.1186/s43556-022-00099-8

**Published:** 2022-12-15

**Authors:** Ruolan Xia, Maosen Xu, Jing Yang, Xuelei Ma

**Affiliations:** 1 Department of Biotherapy, West China Hospital and State Key Laboratory of Biotherapy, Sichuan University, 37 Guoxue Xiang Street, Chengdu, 610041 Sichuan Province China; 2 West China School of Medicine, Sichuan University, Chengdu, China; 3 Melanoma and Sarcoma Medical Oncology Unit, State Key Laboratory of Oncology in South China, Collaborative Innovation Center for Cancer Medicine, Sun Yat-Sen University Cancer Center, 651 Dongfeng Road East, Guangzhou, 510060 China; 4 State Key Laboratory of Oncology in South China, Collaborative Innovation Center for Cancer Medicine, Sun Yat-Sen University Cancer Center, Guangzhou, 510060 P. R. China

**Keywords:** Notch, Hedgehog, Cancer, Cancer stem cell, Tumor microenvironment

## Abstract

Notch and Hedgehog signaling are involved in cancer biology and pathology, including the maintenance of tumor cell proliferation, cancer stem-like cells, and the tumor microenvironment. Given the complexity of Notch signaling in tumors, its role as both a tumor promoter and suppressor, and the crosstalk between pathways, the goal of developing clinically safe, effective, tumor-specific Notch-targeted drugs has remained intractable. Drugs developed against the Hedgehog signaling pathway have affirmed definitive therapeutic effects in basal cell carcinoma; however, in some contexts, the challenges of tumor resistance and recurrence leap to the forefront. The efficacy is very limited for other tumor types. In recent years, we have witnessed an exponential increase in the investigation and recognition of the critical roles of the Notch and Hedgehog signaling pathways in cancers, and the crosstalk between these pathways has vast space and value to explore. A series of clinical trials targeting signaling have been launched continually. In this review, we introduce current advances in the understanding of Notch and Hedgehog signaling and the crosstalk between pathways in specific tumor cell populations and microenvironments. Moreover, we also discuss the potential of targeting Notch and Hedgehog for cancer therapy, intending to promote the leap from bench to bedside.

## Introduction

Notch and Hedgehog (Hh) were first described in Drosophila by Thomas Hunt Morgan in 1917 and by Nusslein-Volhard and Wieschaus in 1980 [1, 2]. Both signaling pathways exist widely in vertebrates and invertebrates, are highly primordial and conserved in evolution, and are intimately involved in the fate determination of cells [3, 4], differentiation of tissues [5], development of organs [2, 6–9], formation of embryos [10, 11] and homeostasis in organisms [12]. Abnormalities in the modulation of both pathways during embryonic development may contribute to malformations [13–15] and disorders [16–19]. Notch signaling has multiple context-specific features and plays an essential role in the maintenance and balance of adult tissues [20]. In contrast, the Hh pathway is predominantly insufficiently active or even quiescent in mature organisms. As necessary, under certain conditions, it would be activated, such as wound healing and tissue recovery [21, 22] in bone [23, 24], intervertebral disc [25], heart, nerves [26–29], skin [30], muscle [31], and gastrointestinal mucosa [32, 33]. However, componential activation of the Hh signaling pathway is responsible for the formation and progression of diverse cancer types, i.e., basal cell carcinoma (BCC) [34, 35]. In addition, an interesting characteristic of Notch signaling is its high sensitivity to dose: both over- and under-activated Notch signaling generate phenotypes, which can partially account for mutations in the Notch pathway that can serve as both carcinogens and suppressors of tumors, relying on the cellular setting [36].

The Notch pathway is an architecturally linear signaling mechanism. Concisely, the intact Notch receptor is a heterodimer made up of an extracellular and a transmembrane subunit. Subsequent interaction with transmembrane ligands presented on the neighboring leads to subunit receptor segregation and causes the transmembrane subunit to be proteolytically hydrolyzed, releasing a Notch intracellular domain (NICD), which is subsequently transported to the nucleus to function as a transcriptional regulator [20, 37]. Pathways in addition to the canonical signaling pathway are also capable of initiating signaling and are categorized as noncanonical NOTCH signaling pathways [38, 39]. Analogously, activation of Hh signaling pathways also proceeds through both canonical and noncanonical routes [40, 41]. The former is activated by the binding of Hh ligand to Ptch1 protein to constitute a complex that relieves the inhibitory effect of smoothened (Smo), followed by a downstream cytoplasmic protein complex consisting of kinesin protein (Kif7), suppressor of fused (SUFU), and glioma-associated oncogene homolog (Gli), which transduces signals to the nucleus and regulates the expression of target genes [42]. Resembling the noncanonical initiation of the Notch pathway, the later activation of Hh signaling can be categorized into three kinds [40]. Nevertheless, contrary to Notch and other embryonic signaling pathways, the Hh signaling pathway is extremely dependent on the primary cilium [43], a single organelle, with the composition of vital signaling components varying in distribution across the cilium depending on whether Hh signaling is switched on or off [44–46].

Within this review, we summarize current advances in the understanding of Notch and Hh biology and crosstalk between pathways in cancer, which will assist in the design of new reasonable therapeutic options [36]. Recent drug development results and efforts to target Notch and Hh signaling are also outlined, and the major categories of investigational drugs thus far described that directly or indirectly alter Notch and Hh signaling are characterized.

## Architecture of the Notch and Hh pathways

### The canonical and noncanonical Notch signaling pathways

**Fig. 1:**
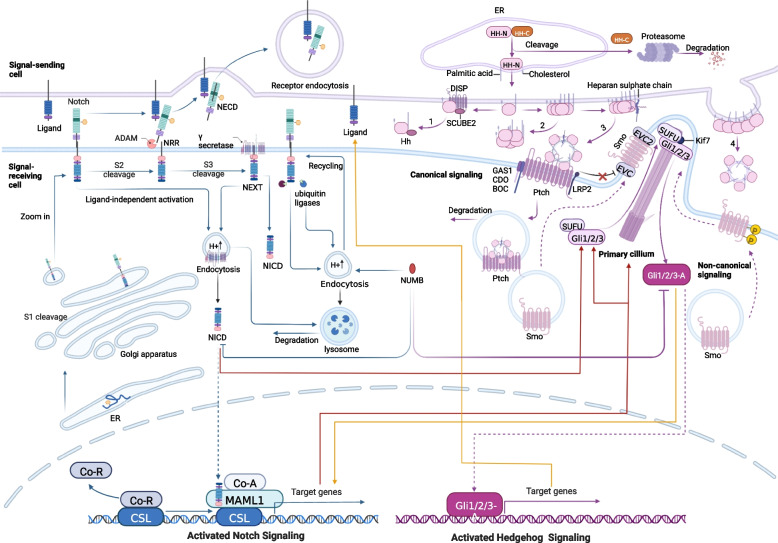
Overview of the Notch and Hedgehog signaling pathways. The blue arrows on the left side of the figure demonstrate the synthesis, modification, transfer and activation of Notch receptor precursors. The purple arrow on the right shows Hh ligand precursor synthesis, modification and translocation, and activation effect of Hh receptors. Crosstalk in the Hh and Notch signaling pathways is shown by red and yellow arrows

Notch signaling is an intercellular communication mechanism initiated by binding between a transmembrane receptor and a membrane-spanning ligand expressed on adjacent cells. The major components and distinct steps in the Notch pathway have been investigated widely and are summarized in brief (Fig. 1) [47, 48].

The canonical Notch signaling pathway is devoid of intermediate links, as the receptor is transduced straight into the nucleus after three cleavage events [12]. The precursor of the Notch receptor is a single-chain protein that is initially transported to the endoplasmic reticulum (ER) after production and extensively glycosylates future extracellular subunits, which modifies the affinity of the Notch receptor for various ligands (orange pentagons represent glycosylation) [49]. The glucosylated precursors are transmitted to the Golgi compartment and undergo the first proteolytic procedures at S1 sites in the Golgi network, yielding noncovalently bound Notch extracellular subunit (N^EC^)-transmembrane and intracellular domain (N^TMIC^) heterodimers to be shipped to the cell membrane as type I transmembrane proteins (NOTCH1-4 for mammals) [50]. The receptor interacts with canonical ligands (DLL1, 4, or Jagged 1, 2) located on juxtaposed cell membranes, exposing a cleavage site that is masked by the LNR domain in the silent phase and arousing dissociation of the receptor subunit by N^EC^ subunit trans-endocytosis into the ligand-expressing cell [51–53]. This unveils the S2 site cleaved by an ADAM 10 or 17 (a disintegrin and metalloproteinase) on the extracellular residue of N^TMIC^ [54]. Subsequent to S2 cleavage, another ligand processing step, the intermediate product of the transmembrane receptor called Notch extracellular truncation (NEXT) [54], is cleaved by the γ-secretase complex at the plasma membrane or in the endosome post-NEXT internalization (S3 cleavage) [55]. This phase releases NICD, which translocates to the nucleus, where it couples with CSL (CBF1/Suppressor of Hairless/LAG1; also named RBPJ), a DNA-binding protein, loosening chromatin [56] and leading to separation of the coinhibitory complex from CSL and facilitating coactivation complex recruitment [47, 57]. The interaction of NICD-CSL is stabilized by Mastermind-like (Maml), a triad of the NICD/CSL/Maml complex that amplifies downstream gene expression [56]. The pool of target genes varies broadly between cell types.

Notch signaling is also initiated by atypical ligands, the deficiency of ligands, the absence of the cleaved Notch receptor, or interactions with other cytoplasmic or cytosolic effectors [38, 39]. It is known as noncanonical Notch signaling, which offers a fascinating new scope for exploration and potentially unmasks new interventional treatment targets. The ligand-independent activation pathway [58] denotes that due to metabolism, ligand-unbound NOTCH receptors can be endocytosed into endosomes containing ADAM and gamma enzymes, triggering S2 and S3 cleavage and thereby activating NOTCH signaling [59, 60]. This pathway is essential for T-cell development [61, 62]. In addition to combining with CSL, NICD crosstalks with signaling pathways such as NF-κB, mTORC, TGF-β, AKT, Wnt, or Hippo at the cytoplasmic or cytosolic level to modulate target gene transcription [63–67]. Classically, reciprocity between NICD and NF-κB affects the properties of multiple malignancies [68–70], suggesting new potential means of blocking noncanonical NOTCH signaling. Moreover, proper ligands and receptors direct cell proliferation, transition and apoptosis in a single/unbound form, and details deserve further elucidation.

Multiple factors act on different components of the pathway involved in regulating Notch signaling. Specific signals, such as AKT, CBFB, SIRT6, RUNX1, and DEC1, control the transcription of receptors that indirectly regulate the entire Notch signaling pathway [71–74]. Certainly, glycosylation of the NOTCH receptors on certain EGF-like repetitive sequences is essential for receptor maturation [75], ligand binding [76, 77], and S2 cleavage [78], and targeting glycosylation is also thought to be effective in inhibiting NOTCH signaling. Intracellular trafficking of receptors mediated by ubiquitination and specific distribution of receptors and ligands across the cell membrane also influence the regional intensity of NOTCH signaling [79, 80]. The balance between activation and degradation following endocytosis is critically associated with downstream signaling. Ligand ubiquitination in signal senders is a requisite for signal activation and mediates ligand endocytosis, facilitating exposure of the negative regulatory region (NRR) domain of the S2 cleavage receptor [81–83]. Notably, cis-inhibition has been observed in many tissues and organisms [84], significantly driving the horizontal suppression process and defining sharp boundaries [85]. Subsequent work is worth extending to explore the potential diversity of signaling states of cells by the combinatorial action of diverse receptors and ligands. Noncoding RNAs likewise occupy a place in the Notch signaling pathway by serving as regulators of respective target genes [86, 87]. Excluding ligands and receptors, the level of Notch signaling can be adjusted by posttranslational modifications of NICD, including hydroxylation, methylation, acetylation, phosphorylation, and sumoylation, which impact the intensity of Notch signaling [88].

### The canonical and noncanonical Hh signaling pathways

The canonical Ptch1-Smo-Gli axis is achieved by autocrine or paracrine patterns. Many excellent publications have previously described in detail the processes that regulate Hh signaling (Fig. 1) [89–91]. Simply speaking, three mammalian counterparts of Hh were found: sonic hedgehog (SHh), Indian hedgehog (IHh) and desert hedgehog (DHh) [92]. Disparities in the timing of expression, spatial distribution and action characteristics among the different ligands were noticed [93]. The maturation and secretion of Hh ligands, glycoprotein precursors synthesized in the ER encoded by a set of vital Hh genes, undergo self-proteolysis, generating C-terminal fragments (Hh-Cs) that are transferred to the proteasome to experience rapid degradation [94], and the N-terminal peptides (Hh-Ns) continue to undergo dual lipidation in the ER by cholesterol and palmitic acid in the C-terminal and N-terminal domains, respectively [95, 96]. The modified Hh-N molecules are attached to the lipid bilayer in monomeric form until they are released by one of four pivotal mechanisms: 1. DISP (a transmembrane transporter-like protein)-mediated and SCUBE2 (a secreted glycoprotein)-coordinated release of Hh-Ns from the cell surface as a monomer [97–100]; 2. Self-aggregation of monomeric HH-Ns to form soluble multimers for liberation [101]; 3. Interaction of HH-N oligomers with heparan sulfate chains of glycoproteins, recruiting apolipoproteins to shape lipoprotein particles [102, 103]; 4. Formation of exovesicle [104]. Spontaneously, canonical Hh signaling is initiated by active Hh ligands. Mature Hh ligands bind to Ptch1/2 [105], a cholesterol transporter enriched in primary cilia (PC). HH-PTC conjugation, facilitated by growth arrest-specific 1 (GAS1), oncogenes (CDO), brother of CDO (BOC), and low-density lipoprotein receptor-related protein 2 (LRP2), induces Ptch to leave the cilium and inactivate, relieving inhibition of Ptch for a seven-pass transmembrane G protein-coupled receptor-like receptor known as Smo. The derepressed Smo subsequently moves to the proximal end of PC to enrich with Ellis‑van Creveld syndrome protein (EVC) and EVC2, suppressing critical protein kinases, including protein kinase A (PKA), casein kinase 1 (CK1), and synthase kinase 3β (GSK3β) [106, 107], depriving the ability to mediate phosphorylation of the SUFU-Gli complex. The role of SUFU as a key suppressor by sequestering Gli in the cytoplasm is dismantled, triggering the detachment of SUFU-Gli and releasing the full-length active Gli (Gli1/2/3-A). Subsequently, the translocation of Gli-A into the nucleus of cells is partially dependent on Kif7 [108], invoking Gli-mediated transcription [109, 110]. Hh target genes consist of Hh signaling components (Ptch, Gli1, GAS1, Hhip), cell cycle regulators (CCND2, CDK) [111, 112], and other transcription factors (VEGFA, PDGFA, BCL2, Wnt, TGF, SNAIL, ELK1, MSX2, BMI1, SOX2, NANOG, Oct4, FOXM1, NR2F1, c-Myc, etc.) [113].

In the absence of Hh ligands, Ptch impedes the transport and localization of Smo in PC [114], leading to phosphorylation and protein cleavage of Gli2 and Gli3 by PKA, CKI and GSK3β to produce repressed forms (Gli2-R and Gli3-R, respectively) [115–119]. Compared to Gli2 and Gli3, Gli1 is primarily regulated at the transcriptional level and is not cleaved into a repressed form. Gli1 can also be manipulated by the ubiquitin‒proteasome system (UPS) [120]. As a result, downstream Hh signaling is aborted. The current Gli pre-patterning model has suffered from challenges, with studies demonstrating that Gli inhibition is not the default state for the Hh pathway [121].

Other than canonical signaling, several pathways activated by Hh ligands have been recognized as "atypical" with no need for PC as a mandatory site [122, 123]. Hh signaling can be classified into three types involving Ptch-Hh-mediated (Type I), Smo-dependent (Type II), and Gli-activated (Type III) signaling according to their modulatory mechanisms [124]. The first type is further divided into two subcategories (Type IA and IB). Ptch1 in Type I can regulate apoptosis by recruiting proapoptotic elements such as caspase-9, dependence-associated receptor transmembrane (DRAL) and tumor-upregulated CARD (caspase-associated recruitment domain)-containing antagonist of caspase nine (TUCAN) to the C-terminal domain. Hitherto, the majority of the noncanonical Hh signaling axis in Type II has been achieved by coupling Smo as a G protein-coupled receptor (GPCR) to a heterotrimeric G protein of the G inhibitory (Gi) family [125, 126], altering several crucial protein kinase activities, including PKA, RhoA, Rac1, NF-1, Src, PI3K/PLCγ, and calcium/calmodulin-dependent protein kinase kinase 2 (CaMKK2) [127–132], and promoting actin rearrangement, immune synapse formation and altered glycolytic capacity [131, 133]. Furthermore, Smo-dependent noncanonical Hh signaling, such as the Gαi-LGN-NuMA-Dynein axis and LKB1/AMPK axis, can facilitate canonical signaling by fostering cilia formation [131]. As observed, various signaling cascades can be involved at the Gli level (Type III), such as RAS-RAF-MEK-ERK [134, 135], PI3K/AKT/mTOR [136–138], TGFβ [139, 140], DYRK family [141], BET proteins, and oncogenic drivers (EWS/FLI1, SOX9 FOXC1, c-MYC) [142–145], which are involved in optimizing Gli activity [146]. Apparently, the activity of Gli can be adversely affected by tumor suppressors (p53, NUMB, SNF5) [147–149], MAPKKK/MEKK [150, 151], and miRNAs (miR-324-5p, miR-361, miR-326) [152–154] in Smo-independent Hh signaling appear to be a fallback activation pathway in canonical Hh signaling and a way for cells to evade the classical Hh pathway to activate downstream gene expression in response to abnormal environmental stress.

### Crosstalk between the Notch and Hedgehog signaling pathways in cancer

Studies have shown that Notch and Hh signaling function synergistically during cell development in several systems, such as the digestive system [155], the nervous system [156], and the immune system [157]. Some studies revealed crosstalk among the Hh, Notch and Wnt pathways that exert synergistic promotive effects in tumorigenesis [158, 159]. The developing nervous system has been more thoroughly studied (Fig. 1). Primarily, it is widely known that Notch signaling can modulate Hh [160]. Second, Hh signaling allows direct or indirect control of Notch through downstream activities [161]. Modulation of the Notch pathway by Hh includes the following: 1. Downstream effectors of Hh govern the transcription of ligands and regulators in the Notch pathway, such as Fringe proteins. 2. Gli proteins deliver immediate, Notch-independent transcriptional regulation of certain Notch target genes [162, 163]. Hh is controlled by Notch signaling in the following ways: 1. Downstream effectors of Notch control the transport of Hh components (e.g., Ptch and Smo) to PC [164]. 2. Downstream effectors of Notch manage Gli levels, irrespective of transcription. 3. NICD plays a direct role in controlling Gli gene transcription [165]. In addition, both Notch and Hh signaling are inhibited by NUMB [148], which contributes to the ubiquitination and degradation of Notch1 through its polypyrimidine tract-binding protein (PTB) structural domain to directly interact with the WW structural domain of Itchy E3 Ubiquitin Ligase (ITCH) [166]. Likewise, NUMB directly inhibits the Hedgehog pathway, while ITCH promotes ubiquitination of Glis1 [167].

Evidence indicates the significance of crosstalk between Hedgehog and Notch in cancer biology and the resistance of cancer stem cells (CSCs) to treatment [168]. However, little is known about the specific molecular mechanisms. A loss-of-function study on Ptch1 showed that it accelerates Notch1-induced T-ALL pathogenesis [169]. In contrast, a mutually exclusive interaction between the Hedgehog and Notch pathways has been found in skin cancer [170]. Notch1 expression deficiency in squamous cell carcinoma correlates with increased Gli2 expression, leading to tumorigenesis [171]. Similarly, the overall activation of Hedgehog and Notch has been described in the etiopathogenesis of pituitary adenoma and prostate cancer [172]. A better understanding and explorations of the regulatory mechanisms of crosstalk between Hh and Notch in the control of tumor onset and progression are needed.

## Role in cancer and therapeutic strategies

### Cancer inhibitor or promoter?

Undoubtedly, Notch signaling exerts bimodal effects in cancer [173]. The homologs of the specific Notch components involved are different in diverse cancers. Gain-of-function mutations involved in Notch genes have been reported in T-ALL [174], splenic marginal zone lymphoma [175], chronic lymphocytic leukemia [176], breast cancer [177], an [178]d adenoid cystic carcinoma [179]. In addition, aberrant activation of wild-type Notch signaling can be observed in lung adenocarcinoma [180], colorectal cancer [181], breast cancer [182], ovarian cancer [183], HCC [184], glioma [185] and other aggressive tumors. Carcinogenic roles of Notch signaling include, but are not limited to, prevention of apoptosis, preservation of a stem cell-like phenotype, induction of epithelial-mesenchymal transition (EMT) [186], generation of drug resistance, promotion of metastasis, facilitation of angiogenesis [187] and mediation of tumor-mesenchymal interactions. In some situations, Notch can manifest as a tumor suppressor, and its loss-of-function or conditional absence results in neuroendocrine tumors [188], squamous cell cancers [189], and pancreatic ductal carcinoma [190]. The mechanisms involved above are complex and context-dependent [173]. Intriguingly, in different subclonal populations within a single tumor, Notch can simultaneously act as both an oncogenic and tumor suppressor [191]. A subpopulation of non-neuroendocrine tumor cells with high Notch activity in mouse small cell lung cancer proliferates more slowly than neuroendocrine cells, in accordance with the tumor suppressive effects of Notch. However, this population of non-neuroendocrine cells is comparatively chemo-resistant and generates growth factors that proliferate neuroendocrine cells with low Notch activity, thus exerting a noncell-autonomous oncogenic effect. Does this suggest that the combination of chemotherapy and Notch inhibitors may be more effective than chemotherapy alone? Less clearly, Notch activity was upregulated in murine-derived HCCs with triple knockout of retinoblastoma protein (RB) and two related RB family members, p107 and p130 [192], but after a pan-Notch blockade was performed, accelerated development of HCC and elevated expression of Notch-related genes were associated with a good prognosis of HCC. This demonstrates that some of the Notch tumor suppressive effects in HCC may not be cell-autonomous, secondary to crosstalk with Wnt in liver-specific tumor-associated macrophages [193]. From the perspective of drug discovery, development, and combination therapy, pan-inhibition or activation of Notch signaling may not be the best strategy; tumor types, target cell populations, Notch paralogs, peripheral immunity, treatment timing, and combination dosing must be considered.

In contrast to Notch signaling, inappropriate activation of Hh signaling leads to carcinogenesis. Deregulation of Hh signaling may be attributed to three key mechanisms: 1. Mutation driver: ligand-independent constitutive amplifications of signaling owing to deactivating mutations in patched receptor (Ptch1) or SUFU [194, 195], invoking mutations in Smo [196] (i.e., BCC [197, 198], subtyped medulloblastoma [199], rhabdomyosarcoma [200]). 2. Ligand dependency: (i) Auto-secretory ligand-dependent activation, in which tumor cells respond to increased expression of their HH ligands in a cell-autonomous manner (i.e., glioblastoma [201], neck squamous cell carcinoma [202, 203], and lung [204], breast [205, 206], ovarian [207], stomach [208, 209], esophageal [210], pancreatic [211], and prostate cancers); (ii) Paracrine-ligand-dependent model, in which Hh ligands produced and released by tumor cells switch on HH signaling in the peripheral stroma, in turn facilitating tumor expansion and metastasis, and creating a positive feedback loop (i.e., prostate, ovarian [212–214], pancreatic [215] and colorectal cancers [216, 217]); (iii) Reverse paracrine ligand-dependent signaling activation, indicates that Hh ligands are secreted from the stroma and received by tumor cells (i.e., multiple myeloma, lymphoma [218], and leukemia [219, 220]). 3. Noncanonical crosstalk: the specific signaling cascade described in the previous mechanism, like RAS-RAF-MEK-ERK and TGFβ [139, 140], upregulates Gli activity and promotes tumorigenesis (i.e., melanoma [221, 222], bladder cancer [223, 224], clear cell renal cell carcinoma [225], and HCC [226]). Although strategies to inactivate the Hh signaling pathway have been intensively studied, contradictory results have been obtained. The existence of interrelationships and cross-talk has pinned hopes for combination therapy. A cell type capable of both self-renewal by symmetric division and of generating more "differentiated" cells by asymmetric division is found in most types of liquid and solid cancers. The field of CSC has been reviewed extensively.

### Cancer stem cells

**Fig. 2:**
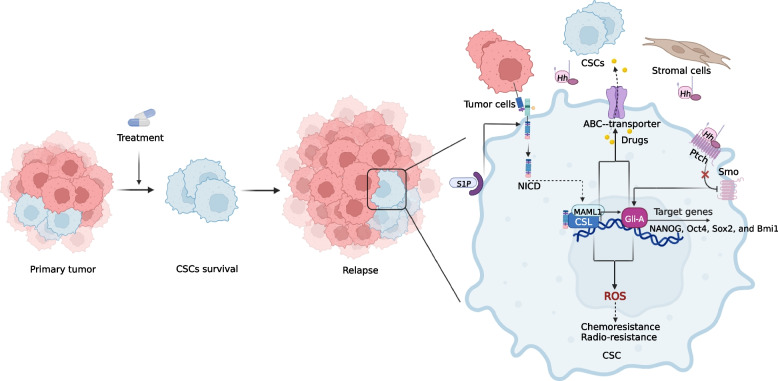
Overview of the Notch and Hedgehog signaling pathways in CSCs. Peripheral tumor cells, CSCs, and mesenchymal cells can initiate the transcription of stemness genes (NANOG, Oct4, Sox2, and Bmi1) in CSCs through Hedgehog signaling to maintain stem cell properties. The role of the Notch and Hedgehog signaling pathways is demonstrated in the zoomed-in CSC on the right side of the figure. S1P enhances CSC activity by binding S1PR secondary to Notch activation. The Hedgehog and Notch pathways also increase ABC-transporters, which increases drug efflux and leads to chemoresistance. The Hedgehog and Notch pathways also increase ROS further affecting the efficacy of subsequent drug and radiation therapy

CSCs, a cell type capable of both self-renewal by symmetric division and of generating more "differentiated" cells by asymmetric division, are found in most types of liquid and solid cancers [227, 228]. and contribute to tumorigenesis, expansion, metastasis, drug resistance and recurrence. Wnt/β-catenin, TGF-β, Hedgehog and Notch are important signaling pathways for the sustainability of self-renewal in CSCs [206, 229](Fig. 2).

Notch signaling plays an essential role in several cancer CSCs. In HER2 + breast cancers, membrane levels of Jagged 1 can be increased by HER2 inhibitors, thereby activating ligand-dependent Notch signaling in CSCs that are resistant to trastuzumab [227]. NOTCH1 activity was enhanced in triple-negative breast cancer (TNBC) CSC population after treatment with rapamycin complex 1/2 inhibitors, suggesting that combination therapy targeting the Notch pathway could be considered for TNBC with mutations leading to PI3K/mTOR pathway activation [230]. In estrogen receptor-positive (ER +) breast cancers, NOTCH1 activity is correlated with the risk of tumor recurrence [231]. More importantly, a number of ER + breast CSCs are NOTCH4 dependent, and elevated NOTCH4 expression relies on phosphorylation of Erα at Ser118 in cell lines containing ERα mutations [232–234]. Therefore, blocking ERα Ser118 phosphorylation or developing targeted Notch4 inhibitors may be valuable tools for the treatment of endocrine-resistant ER + breast cancers. In addition to receptor-associated Notch activation, sphingosine 1-phosphate induces highly oncogenic Notch activation via its own receptor in the ER + breast CSC population, which is also a potential therapeutic target [234]. Indeed, hypoxia, a feature of the CSC microenvironment, triggers Notch activation in glioblastoma CSCs by inducing the production of Vasorin-stabilized membrane-bound NOTCH1, which prevents degradation guided by the endocytic mediator NUMB [235].

Hh signaling is critical for the existence of subpopulations of tumor cells that exhibit stem cell characteristics [236, 237]. Hh signaling enables CSCs to maintain a stemness profile in multiple cancers by driving the expression of stemness-regulated genes such as NANOG, Oct4, Sox2, and Bmi1 [238–240]. CD200 + CD45 cancer cells have been determined to be a CSC population in BCC that expresses high levels of Gli1 and depends on Gli1 for survival [240]. Combination therapy with anti-CD200 + -neutralizing antibodies and Notch inhibitors is a new route to eradicate BCC. Noncanonical Ptch1-dependent Hh signaling is required for the survival of colon CSCs, particularly its regulation with crosstalk of the Wnt/β-catenin signaling pathway [241]. Suppression of Hh signaling was shown to inhibit pancreatic CSC growth in both in vitro and in vivo models, reversing the chemoresistance and stemness acquired by pancreatic cancer cells to the administration of gemcitabine [242]. The important role of Hh-Notch crosstalk in CSCs concerning cancer biology and chemoresistance has been confirmed, with conserved oncogenic properties associated with hypoxia and immunoevasion [243].

### Migration and metastasis

Invasion and metastasis are unique properties of malignant tumors, and Notch and Hh are intertwined with other pathways that control tumor heterogeneity, stemness, EMT, angiogenic tumor cell dormancy, and other behaviors associated with tumor invasion and distant metastasis. Notch can interact directly or indirectly with ECM components [244]. In addition, cancer cell invasion into blood or lymphatic vessels relies on an actin-based structure called invadopodia that can be formed by Notch signaling mediated by activation upon hypoxia [245]. The coordinated response between NOTCH and EMT provides support for local angiogenesis and lymphangiogenesis [246, 247]. It is significant for the formation of circulating tumor cell (CTC) clusters, stabilization of CTCs in the bloodstream and extravasation of CTCs [248–251]. Several relatively isolated studies have also indicated a potential role of Notch in metastasis. Studies targeting Jagged ligands, their upstream regulators, or downstream effectors have proven that a high level of Jagged 1 is tightly associated with metastasis and recurrence of osteosarcoma [252]. Furthermore, TGFβ increases Jagged 1 expression in metastatic breast cancer cells, and Jagged 1 activates NOTCH1 in osteoblasts, causing IL-6 expression to support the survival of metastatic breast cancer cells and leading to the development of osteoclasts mediating bone breakdown. In chemotherapy-induced heterogeneous conditions within breast cancer, cancer cells evolve EMT by enhancing Notch signaling and have a greater ability to migrate and invade [253]. The relationship between Jagged-mediated Notch signaling and metastasis can also be observed in prostate cancer. Li et al. identified a novel FAS-ERK-JAG1-NOTCH1 axis that may be involved in the stemness and lung metastasis of oral squamous carcinoma [254]. In HCC, NET-4 promotes cell migration and tumor metastasis by increasing the enzymatic maturation of ADAM10, which in turn increases Notch signaling activated by cleavage of the Notch1 receptor catalyzed by the γ-secretase complex [255]. Activation of the Jagged-1/Notch/CXCR4 axis is also relevant to specific tumors [256]. NOTCH3 levels have also been shown to be associated with TNBC seeding and metastasis. Phosphoserine aminotransferase 1-specific upregulation intervenes in the metastasis of ER- breast cancer by prolonging the half-lives of NICD1 and β-catenin [257]. HOX transcript antisense intergenic RNA can positively correlate with the regulation of the Notch signaling pathway associated with tongue cancer invasion and metastasis [258].

Compared to invasion and metastasis with Notch, Hh has been relatively poorly studied and poorly linked. A number of studies revealed that high Hedgehog-Gli characteristics are associated with metastasis in colon cancer [119, 259, 260]. Gli1 activity is relevant to tumor aggressiveness in papillary thyroid carcinoma. Hedgehog promotes EMT in a variety of solid tumors, including liver, pancreatic, colon, and breast cancers [261, 262]. Shh enhanced the locomotion and invasiveness of gastric and ovarian cancer cells, while no assistance was observed in cells treated with anti-Shh monoclonal [263, 264]. The growth, adhesion and migration of lung adenocarcinoma cells are associated with activation of the PI3K and SHh pathways [265]. Degalactotigonin suppresses human osteosarcoma metastasis by regulating GSK3β inactivation-mediated inhibition of the Hedgehog/Gli1 pathway [266]. Circ-Gli1 indirectly upregulated Cyr61 through the activation of the Hedgehog and Wnt pathways, thereby exacerbating melanoma metastasis and angiogenesis. In addition, circ-Gli1 induced GSK3β phosphorylation at Ser9 under certain conditions, preventing GSK3β from binding to Gli1 and β-catenin, thereby increasing Gli1 and β-catenin protein expression [267].

### Treatment resistance and tumor recurrence

The prominent problem we face with current anticancer therapies, including chemotherapy, radiotherapy, targeted therapy and immunotherapy, is the emergence of drug resistance leading to the ineffective control of tumors. The mechanisms of drug resistance are often multifaceted and complicated, including altered drug transport, drug metabolism, altered drug targets, blockade of apoptosis, alteration of the cell cycle, formation of EMT, induction of the CSC phenotype, alteration of the tumor microenvironment (TME), etc [268, 269].

For chemotherapy, Notch and Hh signaling adjust the expression of membrane transporters to regulate drug efflux. Upregulation of Notch by doxorubicin in prostate and breast cancers increased the expression of adenosine triphosphate-binding cassette subfamily B member 1 (ABCB1) and drug resistance-associated protein 1 (MRP1), which increased drug efflux and led to chemoresistance [270, 271]. This chemo-resistant effect is reversed by Notch inhibitors. Notch controls apoptosis, which is reflected in ovarian cancer, pancreatic cancer, HCC, osteosarcoma and glioblastoma that are resistant to chemotherapy, and inhibition of Notch activity leads to apoptosis [272–275]. Notch controls the cell cycle by inducing the expression of several key cell cycle-related genes to develop drug resistance [276]. Notch affects chemoresistance by controlling reactive oxygen species (ROS) levels in CSCs [277]. Similarly, Hh directly modulates ABCB1 and ABCG2 expression, and inhibition of Gli1 improves the response to chemotherapeutic agents in ovarian cancer [278]. Inhibitors of Gli in combination with temozolomide for glioblastoma induce apoptosis in already resistant cells [275]. Hh delivers ROS-induced signaling to regulate chemoresistance in various cancers. ROS-associated activation of NRF2 upregulates SHh, leading to sorafenib resistance in HCC [279]. Drug resistance associated with EMT, CSCs and the TME is also closely linked to both pathways [280, 281]. Activation of SHh promotes migration and invasion and drives DDP resistance in bladder cancer [282].

In radiotherapy, Notch signaling is initiated upon irradiation and regulates DNA damage repair by directly reacting with ATM and disabling its kinase activity [283, 284]. Notch signaling can also influence radio-resistance by mediating the function of other molecules involved in cell survival, metabolism, ROS, and the cell cycle. In glioma CSCs, Notch inhibition does not change the DNA damage reaction in CSCs but inhibits Akt and Mcl1 activities after radiation, making CSCs more sensitive to radiation [285]. Notch signaling mediated by NRF2 associated with cellular oxidation has been an important determinant of radio-resistance in lung cancer cells. Notch signaling mediated by NRF2 associated with cellular oxidation has been an essential decision factor for radio-resistance in lung cancer cells. The role of Hh in tumor radio-resistance has also been mentioned. Gli1 is usually upregulated at the HNSCC tumor-stroma crossover after irradiation and assists in stroma-mediated radio-resistance, which can be reduced by pharmacological inhibition [286, 287].

In terms of targeted therapies, tumors resistant to tyrosine kinase inhibitors (TKIs) frequently exhibit Notch upregulation. In AML with FLT3 mutations treated with FLT3-TKI, Notch signaling is upregulated, leading to alternative ERK activation and resistance. This resistance can be eliminated by Notch inhibitors [288]. Similarly, resistance to TKIs in lung adenocarcinomas with EGFR mutations and to BRAFi in melanomas carrying activated BRAF mutations can be eliminated by Notch inhibitors [289, 290]. The interaction of Hh with the EGFR signaling pathway is evident in many embryonic developmental processes and is therefore relevant to the development of TKI resistance. NSCLC and HNSCC cells with EGFR-TKI resistance exhibit hyperactivation of Hh signaling [291]. Hh pathway inhibitors act synergistically with TKIs to increase tumor sensitivity to chemotherapy and prolong survival in tumor-bearing animals [292]. To date, several mechanisms of resistance to Smo antagonists targeting the Hh pathway have been successively uncovered, including the following. Hh pathway components include Smo mutations [293, 294], SUFU deletion [295, 296], and amplification of Gli or Hh target genes [296]. 2. activation of nonclassical Hh pathways [141, 297, 298]. 3. loss of primitive cilia [299].

In the context of immunotherapy, Notch signaling has a regulatory role in immune cells in the local or systemic TME, and activation of Notch facilitates immunotherapy [300]. A significant association between NOTCH1/2/3 mutations and better outcome with immune checkpoint inhibitors (ICIs) was found in wild-type genetic NSCLC patients [301]. Hh affects immune responses in a complicated and varied manner. Hh inhibitors in pancreatic ductal adenocarcinoma suppress cancer-associated fibroblast (CAF) proliferation and translation and diminish the barrier to immune cell infiltration into the tumor [302]. Tumor cell-secreted Shh induces tumor-associated macrophage (TAM) M2 polarization and restrains CD8 + T-cell recruitment to the TME, thereby mediating immunosuppression [303]. Hh activity is not only a predictor of resistance to ICIs as a biomarker, but in association with PD-L1 expression, it can better forecast clinical outcomes [304].

The generation of resistance to anticancer therapies is a major cause of failure of various treatments. Tumors evolve through these conserved developmental signaling pathways (Notch, Hh) under the selective pressure of therapy, resulting in drug resistance. Therefore, the exploration of these pathways is necessary to eradicate drug resistance.

## Tumor microenvironment

Recent evidence suggests that there are intricate interactions between tumor cells and stroma that have a significant impact on both tumor progression and regression. In particular, the mutual interactions between tumor cells and immune cells and CAFs deserve deeper and more exploration.

### Cancer-associated immune cells

**Fig. 3:**
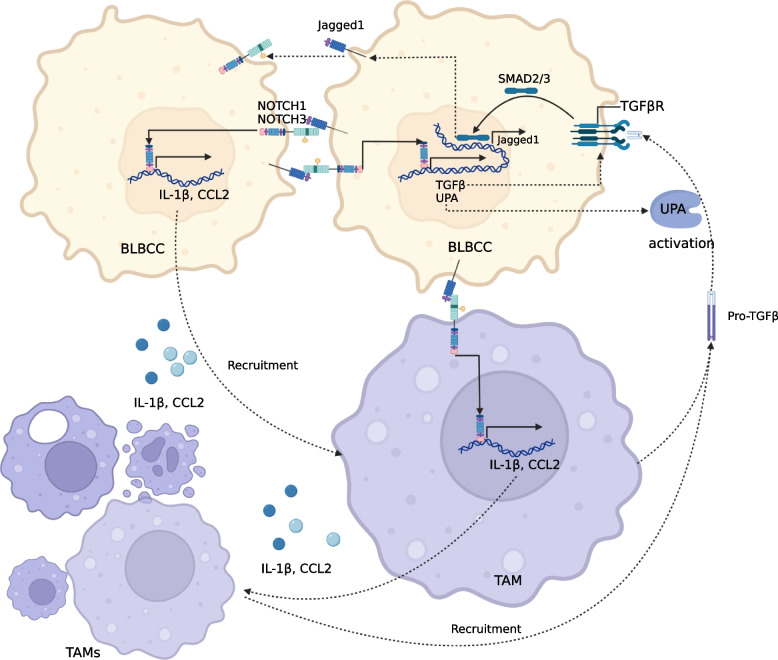
Notch-mediated paracrine cycle between BLBCC and TAMs. Activated NOTCH1 and NOTCH3 (possibly) upregulate the expression of IL-1β and CCL2 in tumor cells. IL-1β and CCL2 recruit monocytes into the tumor microenvironment to mature into TAMs. TAM-secreted IL-1β and CCL2 recruit more monocytes and TAMs additionally secrete TGFβ-pro. TGFβ-pro is activated by UPA After being TGFβ, it induces Jagged 1 expression in cancer cells through its receptor TGFβR1 in combination with SMAD2/3. Jagged 1 activates NOTCH1 and NOTCH3 (probably), thus upregulating IL-1β and CCL2 expression

Notch and Hh signaling play an important role in immune system development and homeostasis. The roles of Notch and Hh in immune cells have attracted much attention for immunotherapy reasons. Notch has been demonstrated to play a dual sword role in the regulation of immune responses in tumors, a role equal to that of regular hematopoiesis and the production of immune effector cells [305](Fig. 3). Upregulation of NOTCH1 and NOTCH3 (perhaps) in basal- like breast cancer cell(BLBCC) leads to secretion of cytokines, such as IL1β and CCL2, which recruit monocytes that mature into tumor-associated macrophages (TAMs) in stroma. TAM can secrete TGFβ, which combines with the receptor TGFβR1 in tumor cells to induce Jagged 1 via SMAD2/3. TAM can secrete TGFβ, which combines with the receptor TGFβR1 in tumor cells to induce Jagged 1 via SMAD2/3. Jagged 1 in turn induces NOTCH1 and perhaps NOTCH3, forming a Jagged-involved tumor-stromal paracrine signaling loop [306, 307]. Myeloid-derived suppressor cells (MDSCs) enhance the stemness of breast cancer cells by activating Notch and STAT3 [308]. Dysregulation of Notch signaling in immature T cells induces MDSCs in T-ALL-bearing mice [309]. The absence of NOTCH2 in CD8 + T cells compromises the antitumor response, which can be enhanced by Notch stimulation of DLL1-expressing dendritic cells [310–312]. An additional potential function of DLL1-mediated Notch activation is to coculture with activated CD4/8 + T cells to convert them into stem cell-like memory T cells, which upon restimulation leads to a positive antitumor response [313]. Jagged1-induced Notch stimulation increases the expression and secretion of multiple cytokines associated with increased macrophage infiltration and reduced T-cell activity [314]. High NOTCH3 expression is associated with low infiltration of CD8 + T cells and high infiltration of immunosuppressive cells in gastric cancer [315]. The Mechanism section mentions TCR-mediated ligand-independent Notch activation, the intensity of which is determined by the amount of NOTCH1 and NOTCH2 (possibly) interacting with the immune synapse [61]. Conditional expression of the intracellular domain of NOTCH1 under the granzyme B promoter in CD8 + T cells with antigen specificity enhanced the cytotoxic response and overcame the tolerogenic effect of MDSCs. This provides new insight into the enhanced anticancer activity of chimeric antigen receptor (CAR) T cells [316].

Hh signaling facilitates the activation of lymphocyte functions such as migration, multiplication, and cytotoxicity [317]. However, Hh was previously proven to promote TAM polarization to restrain tumor-infiltrating CD8 T + cell recruitment (mentioned in the drug resistance section) [303]. Hh also promotes Th2 differentiation in naive human CD4 + T cells [318]. Gli1 evokes polarization of invading myeloid cells toward MDSCs by inducing Schlafen 4 [319]. Hh signaling is also implicated in the functions of dendritic cells under hypoxic conditions, including migration, chemotaxis, phagocytosis, and cytokine secretion [320]. The above results suggest that the up- or downregulation of immune cell function and immune response are dependent on Hh signaling.

### Cancer-associated fibroblasts

CAFs are essential components of the TME, yielding and remodeling an extensive extracellular matrix, accounting for a substantial proportion of the tumor volume [321, 322]. Conventional fibroblasts are an important source of CAFs, and other cell types may play a partial role [323]. Notch is considered to function in CAF activation and in crosstalk between CAFs and mutant cancer cells. In ductal carcinoma in situ (DCIS) of the breast, direct Notch-mediated crosstalk between tumor cells and CAFs was found [324, 325]. Direct interaction between Jagged1 on breast DCIS and NOTCH2 receptors on peripheral fibroblasts is possible [326]. In lung cancer, Notch activation is induced in CAFs and is associated with poor prognosis [327, 328]. Myofibroblastic CAFs (myCAFs) may be activated by IL-6/IL-8-mediated Notch/HES1 and STAT3 pathways in the tumor microenvironment to increase CSCs [329]. Notch modulates two matrix-degrading enzymes, MMP2 and MMP4, via NF-κB signaling to remodel the extracellular matrix in the TME [330]. Similarly, urokinase PA can be directly regulated by Notch [331].

CAFs are not only a potential source of Hh ligands in the cancer stroma but may also respond to H signaling through nuclear Gli-1 activation [332]. However, it has been mentioned in reviews that CAFs that inhibit tumor progression through stroma-specific Hh stimulation have been detected in cancer-bearing mice, including tumor models of the colon, bladder, and pancreas [333–335]. Inhibition of Hh reduced the number of myCAFs and increased the number of inflammatory CAFs in pancreatic ductal adenocarcinoma, which correlated with a cytotoxic T-cell reduction and regulatory T-cell increment, consistent with increased immunosuppression [336]. Given the heterogeneity of CAFs, a number of studies have shown that CAF-mediated Hh signaling is biased toward pro-tumorigenicity. SHh is highly expressed in CAF lysate CAF-derived exosomes, improving the growth and migration ability of esophageal cancer cells in vitro and in vivo. In addition, CAFs secreted extracellular vesicle-encapsulated miR-10a-5p, which activated Hh signaling and promoted angiogenesis and tumorigenicity in cervical squamous cell carcinoma cells [337]. In prostate cancer, CAFs expressing high levels of CD90 play a key role in promoting tumor progression by activating Hh and altering other signaling pathways [211].

### Tumor angiogenesis

**Fig. 4:**
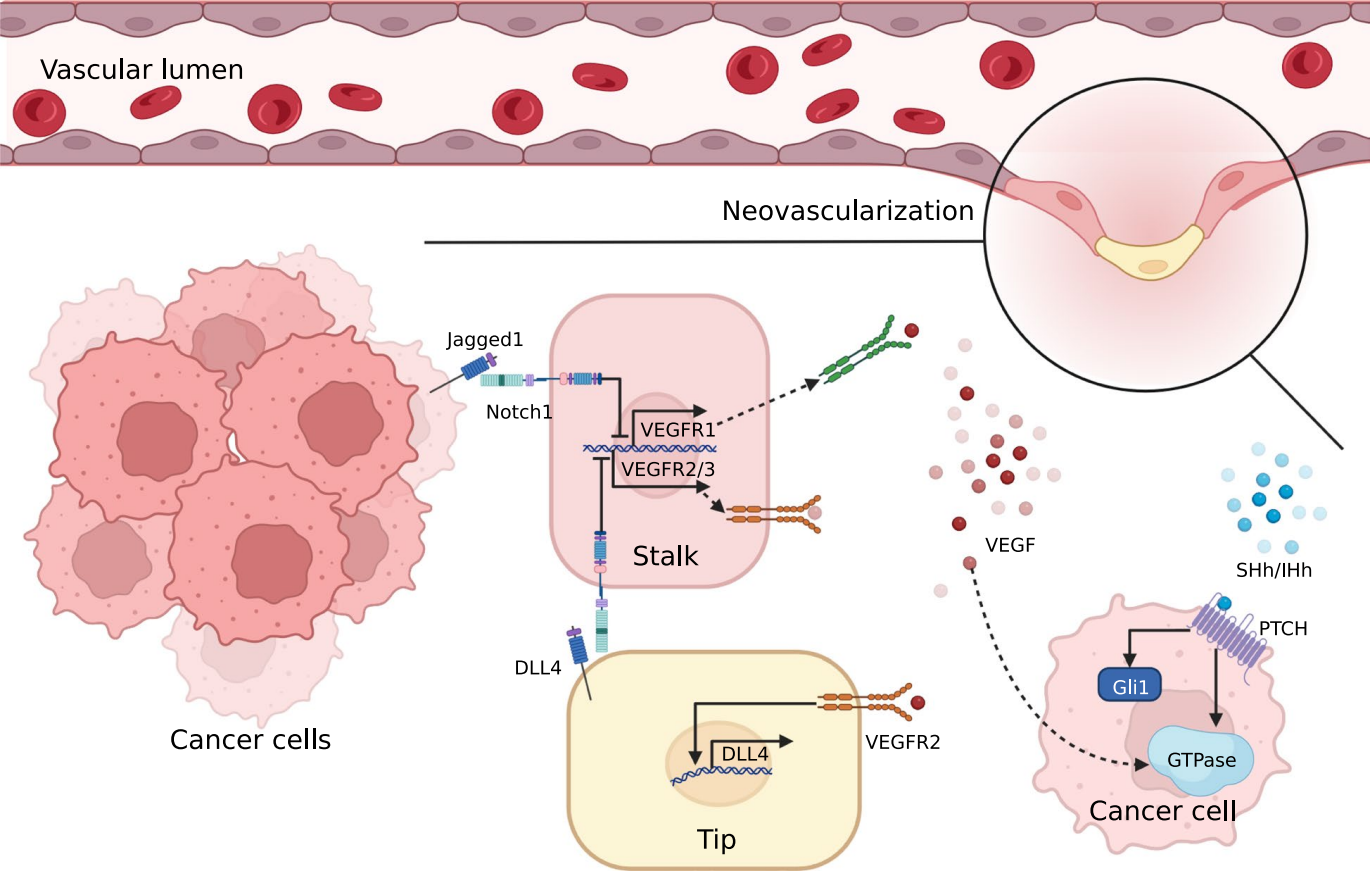
Notch- and Hh-mediated tumor neovascularization

Control of angiogenesis is considered a promising approach to limit tumor progression and metastasis (Fig. 4). During physiological angiogenesis, Activation of Dll4-Notch-VEGFR2 axis is essential for appropriate angiogenesis and couples germinating angiogenesis to arteriogenesis. Similarities are shown in tumorigenic angiogenesis, activation of VEGF signaling in the "tip cells" at the front of the new vascular buds induces the expression of DLL4, which subsequently triggers the activation of NOTCH1 in adjacent endothelial cells and suppresses the expression of VEGFR2 and VEGFR3 in adjacent endothelial cells, giving the cells a "stalk" phenotype and stopping the sprouting of new capillary vessel, and eventually forming new vessels [338]. Blockade of DLL4 causes vascular hypersprouting and disturbed angiogenesis. mAbs of DLL4 are effective in preclinical models of cancer [339]. Nevertheless, chronically inhibiting DLL4-NOTCH1 signaling induces hemangiomas and hemorrhages. Besides, Jagged 1 expressed by tumor cells mediates Notch activation and inhibits the expression of soluble VEGFR1, which traps VEGF outside the cell and antagonizes VEGF signaling activation [340]. It is suggested that selective intervention with Jagged 1 is an appealing potential strategy for regulating tumor angiogenesis.

The Hh pathway has been implicated in vascular development and neovascularization in human embryonic development, tissue injury and disease [341–343]. Indeed, the role of the Hh pathway in modulating tumor angiogenesis still deserves to be further explored [344]. There is a significant association between IHh and VEGF protein expression in HCC [345]. In pancreatic cancer, SHh indirectly promotes tumor angiogenesis by inducing Ptch1 and Gli1, thereby inhibiting two enriched stroma-derived antiangiogenic factors (THBS2 and TIMP2), and directly promotes tumor angiogenesis by activating small GTPases of the RHO family in the presence of VEGF [346]. Analogously, inhibition of nuclear translocation of Gli1 and transcriptional activity associated with Hh signaling can also inhibit angiogenesis in TNBC [347]. Moreover, extracellular vesicle-encapsulated microRNA-10a-5p detached from CAFs promotes cervical squamous cell carcinoma cell angiogenesis via the Hh signaling pathway [337].

## Targeting Hedgehog and Notch signaling

Given the critical roles of Notch and Hh signaling in malignant transformation and oncogenic advancement, several agents targeting these pathways have been designed to potentiate antitumor effects and have been tested in animal models. An increasing number of targeted drugs have also been advanced to testing in clinical trials. In this section, we summarize current strategies targeting Notch and Hh signaling and discuss their anticancer effects, with the aim of promoting the leap from bench to bedside.

### Therapeutic strategies based on Notch signaling

In light of the aberrant expression and wide participation of Notch signaling in tumor development, widespread efforts have been made to search for Notch-targeted therapeutic approaches, and numerous agents are being investigated in preclinical studies and undergoing clinical trials for cancer treatment [348, 349]. Current targeted strategies tested in the clinic principally converge on inhibiting or regulating γ-secretase and blocking the ligand‒receptor interaction via related monoclonal antibodies (Table 1) [350].

**Table 1:** Recent clinical trials targeting the Notch pathway

Target	Drug name	Conditions	Combination	Phase	Current states	Trial number
γ-Secretase	LY3039478 (JSMD194)	T-ALL/T-LBL	Dexamethasone	I/II	Completed	NCT02518113
		Advanced Cancer	Prednisone	I	Completed	NCT01695005
		Advanced or Metastatic Solid Tumors	Taladegib, Abemaciclib, Cisplatin, Gemcitabine, Carboplatin, LY3023414	I	Completed	NCT02784795
	Nirogacestat (PF-03084014)	Desmoid Tumors / Aggressive Fibromatosis	-	II	Active, not recruiting	NCT01981551
		Advanced Breast Cancer	-	II	Terminated	NCT02299635
		Advanced Cancer And Leukemia	-	I	Completed	NCT00878189
		Progressive, surgically unresectable desmoid tumors	-	II	Recruiting	NCT04195399
		Advanced Breast Cancer	Docetaxel	I	Terminated	NCT01876251
		Metastatic Pancreatic Adenocarcinoma	Gemcitabine and Nab-paclitaxel	II	Terminated	NCT02109445
		Desmoid Tumors	-	III	Active, not recruiting	NCT03785964
	RO4929097 (R4733)	Breast Cancer	Carboplatin, paclitaxel	I	Terminated	NCT01238133
		Metastatic Pancreas Cancer	-	II	Completed	NCT01232829
		Advanced Solid Tumors	Ketoconazole, rifampin, Midazolam, Hydrochloride, Omeprazole, Tolbutamide and Dextromethorphan Hydrobromide	I	Completed	NCT01218620
		Metastatic Colorectal Cancer	-	II	Completed	NCT01116687
		Melanoma	-	II	Terminated	NCT01120275
		Metastatic Epithelial Ovarian Cancer, Fallopian Tube Cancer, or Primary Peritoneal Cancer	-	II	Completed	NCT01175343
		Glioblastoma	-	II	Terminated	NCT01122901
		Advanced Solid Tumors	-	I	Completed	NCT00532090
		Malignant Glioma	Temozolomide and radiation therapy	I	Completed	NCT01119599
	AL101 (BMS-906024)	Breast cancer	-	II	Active, not recruiting	NCT04461600
		Adenoid cystic carcinoma	-	II	Active, not recruiting	NCT03691207
		Advanced or Metastatic Solid Tumors	-	I	Completed	NCT01292655
		Acute T-cell Lymphoblastic Leukemia or T-cell Lymphoblastic Lymphoma	Dexamethasone	I	Completed	NCT01363817
		Advanced / Metastatic Solid Tumors	Paclitaxel, 5-Fluorouracil, Carboplatin, Leucovorin, Irinotecan	I	Completed	NCT01653470
DLL3	Rovalpituzumab tesirine (Rova-T)	Small cell lung cancer	Topotecan, Dexamethasone	III	Completed	NCT03061812
		Small cell lung cancer	Dexamethasone	III	Terminated	NCT03033511
		Small cell lung cancer	-	II	Completed	NCT02674568
		Cancer	-	II	Completed	NCT03543358
		Small cell lung cancer	-	I/II	Completed	NCT01901653
		Small cell lung cancer	Cisplatin, Etoposide	I	Terminated	NCT02819999
		Small cell lung cancer	Ipilimumab, Nivolumab	I/II	Terminated	NCT03026166
	AMG757	Small cell lung cancer	Pembrolizumab, CRS Mitigation Strategies	I	Recruiting	NCT03319940
	HPN-328	Small cell lung cancer	-	I/II	Recruiting	NCT04471727
DLL4	Enoticumab (REGN421)	Advanced Solid Malignancies	-	I	Completed	NCT00871559
	Demcizumab (OMP-21M18)	Non-Squamous Non-Small Cell Lung Cancer	-	I	Completed	NCT01189968
		Platinum Resistant Ovarian	Taxol	I	Terminated	NCT01952249
		Locally Advanced or Metastatic Pancreatic Cancer	Abraxane, gemcitabine	I	Completed	NCT01189929
		Metastatic Pancreatic Ductal Adenocarcinoma	Abraxane, gemcitabine	II	Completed	NCT02289898
		Non-Squamous Non-Small Cell Lung Cancer	Pemetrexed, carboplatin	II	Completed	NCT02259582
		Locally Advanced or Metastatic Solid Tumors	Pembrolizumab	I	Completed	NCT02722954
Notch 1	Brontictuzumab (OMP-52M51)	Solid Tumors	-	I	Completed	NCT01778439
		Adenoid Cystic Carcinoma	-	-	Completed	NCT02662608
		Lymphoid Malignancies	-	I	Completed	NCT01703572
		Metastatic Colorectal Cancer	Trifluridine/tipiracil	I	Completed	NCT03031691
Notch 2/3	Tarextumab (OMP-59R5)	Pancreatic Cancer	Gemcitabine, Nab-Paclitaxel	I/II	Completed	NCT01647828
		Small Cell Lung Cancer	Etoposide, Cisplatin or Carboplatin	I/II	Terminated	NCT01859741
		Solid Tumors	-	I	Completed	NCT01277146
Notch 3	PF-06650808	Advanced solid tumors	-	I	Terminated	NCT02129205
Pan- Notch (transcriptional complex)	CB-103	Advanced or metastatic solid tumors and haematological malignancies	-	I/II	Recruiting	NCT03422679

The relevant clinical trial data were obtained from the registration on ClinicalTrials.gov

#### γ-Secretase inhibitors and modulators

Originally developed and used for the treatment of Alzheimer’s disease, γ-secretase inhibitors (GSIs) are currently extensively studied as anticancer drugs due to their potential to inhibit the activation of Notch signaling [351, 352]. In preclinical tumor models, GSIs have shown antitumor effects in a variety of tumor models, including lung [353], breast [354], glioma [355], pancreatic [356], colorectal [357], and ovarian cancers [358]. Although the application of pan-Notch GSI sometimes results in unexpected toxicity [359, 360], its antitumor activity has been shown in many cancer types through diverse mechanisms [361–364]. These promising findings motivated the initiation of a series of phased clinical trials. However, some of the GSIs (BMS-986115 [365], RO4929097 [366], MK0752 [367]) were discontinued in phase I clinical trials due to dose-limiting toxicity. Only RO4929097 and PF-03084014 have been included in phase II trials to date. Regrettably, the antitumor effects achieved were more limited, with no objective remission responses observed in the treatment of platinum-resistant ovarian cancer with RO4929097 [368]. Efficacy was also poor in metastatic melanoma [369], glioblastoma [370], and metastatic colorectal cancer [371]. Promisingly, PF-03084014 (nirogacestat) in desmoid tumors allowed 29% of patients to undergo a partial response lasting more than 2 years [372]. A phase III clinical trial of nirogacestat is expected. In contrast to the paninhibitory effect of GSIs on γ-secretase, γ-secretase modulators (GSMs) maintain a portion of Notch signaling function by altering the catalytic activity of γ-secretase [373]. Treatment of T-ALL cell lines with MRK-560 targeting the catalytic subclass of the γ-secretase complex has been demonstrated to decrease mutant NOTCH1 processing and lead to cell cycle arrest, and the toxicity can be tolerated [374].

#### Antibodies against ligands and receptors

Given that the location of extracellular ligands and receptors facilitates direct drug transport and targeted binding, enabling the development of antibodies targeting receptors and ligands was considered [375, 376]. Targeting antibodies against different receptors and ligands enables precise targeting and reduces serious adverse effects.

#### Antibodies against ligands

For the Notch ligands currently being explored and discovered, Jagged1, DLL3 and DLL4 are the most relevant and good quality studies. At the preclinical stage, 15D11 is one of the most prospective human monoclonal antibodies targeted against Jagged 1, with improved chemosensitivity, reduced bone metastases, and a high safety profile [377]. High expression of both Jagged 2 and DLL1 is positively associated with tumor progression and may also be promising targets, although agents aimed at these ligands have not been reported [328, 378]. DLL3 is an inhibitory ligand for NOTCH and is one of the ligands with positive results in clinical applications [379]. Rovalpituzumab tesirine (Rova-T) results revealed an objective remission rate of 18.3% and a 38% controlled incidence of severe drug-related AEs in patients with SCLC and large cell neuroendocrine carcinoma (LCNEC) [376]. Rova-T has a shorter OS and lower safety rate than standard second-line chemotherapy [380]. The mOS for SCLC patients receiving Rova-T after at least two lines of treatment was only 5.6 months, with an ORR of 12.4% [381]. Two clinical trials on SCLC, AM757 (bispecific antibody) and HPN328 (trispecific antibody), are still under recruitment. In addition, patient-derived glioma tumor spheroids with IDH mutations are susceptible to Rova-T in vitro, implicating the possibility of further exploration of indications and new drugs [382]. DLL4 is an important promoter of tumor angiogenesis and maintenance of CSCs [339]. It exhibits antitumor effects in ovarian cancer when blocked in combination with VEGF [383]. In phase I clinical studies, solid tumors exhibited a therapeutic response to both Enoticumab and Demcizumab, but the latter was associated with significant cardiotoxicity [384, 385]. Targeting DLL4 and VEGF dual variable domain immunoglobulins, ABT-165 demonstrated excellent efficacy and safety in preclinical models, and navicixizumab has shown modest antitumor efficacy and AEs in a phase I clinical trial in solid tumors [386, 387].

#### Antibodies aimed at receptors

NOTCH1 serves as a facilitator in various tumors, such as colorectal cancer and T-ALL, and is regarded as a possible antitumor target [388, 389]. In a phase I clinical trial for refractory solid tumors, the administration of brontictuzumab resulted in partial response in 2 patients and stable disease at ≥ 6 months in 4 patients [390]. Similarly, NOTCH2 and NOTCH3 act as promoters in breast cancer, pancreatic ductal adenocarcinoma [391], and lung cancer [392]. OMP-59R5, a NOTCH2 and NOTCH3 blocker, has been investigated in PDAC [393], SCLC, and other solid tumors in clinical trials [394]. However, OMP-59R5 in combination with chemotherapy has not achieved a good objective therapeutic response. PF-06650808 is a novel anti-NOTCH3 ADC that has achieved remission with a controlled safety profile in breast cancer patients with positive NOTCH3 expression [395]. The effect of NOTCH4 on tumors is context dependent, and there is a lack of well-studied agents.

#### ADAM inhibitors

ADAM10, or in some cases ADAM17, is the key protein for S2 cleavage of the ligand‒receptor binding domain in the Notch pathway. A phase I clinical trial of the bispecific ADAM10/17 inhibitor INCB7839 (aderbasib) is being conducted in recurrent, high-grade pediatric gliomas [396]. ZLDI-8, a novel ADAM17 inhibitor, was found to inhibit Notch and reverse rectal cancer resistance to 5-fluorouracil or irinotecan in vivo and in vitro [397]. Inhibitors of specific ADAM10 have not been reported.

#### Transcriptional complex inhibitors

Activation of transcription is the final step in signaling. NOTCH transcription is dependent on a complex composed of CSL, NICD, and Maml. The small molecule IMR-1 disrupts the recruitment of Maml1 and attenuates target gene transcription [398]. CB103 is described as an inhibitor of the Notch transcriptional complex via protein–protein interactions and is currently in clinical development with initial success in the inhibition of breast cancer and leukemia xenografts [399, 400].

### Targeting Hh signaling for cancer therapy

It was elucidated that Hedgehog signaling could be blocked at various sites. Over the past decades, different methods aiming to inhibit the Hh pathway have been widely investigated in both solid and hematological cancers. Current targeted strategies mainly focus on inhibiting key components in the pathway cascade, such as SHh, Smo, and Gli [401–404].

#### Targeting SHh

The overexpression of SHh contributes to excessive Hh signaling and is positively correlated with tumor burden in nearly all cancer types [405]. As such, inhibiting SHh may represent an attractive therapeutic opportunity. Some SHh inhibitors have been developed to delay tumor progression. For example, 5E1 is a monoclonal anti-Shh antibody that can block the binding of SHh from interacting with Ptch1. The use of 5E1 was demonstrated to repress tumor proliferation and enhance the efficacy of radiation and cisplatin in cervical cancer [406–408]. RU-SKI 43, a dihydrothienopyridine derivative that was designed to target Hh acyltransferase [409], and previous studies confirmed its antitumor potential both in vivo and in vitro [410, 411]. Sulforaphane is the main ingredient in broccoli/broccoli sprouts and was recently found to inhibit leukemia stem-like cell growth and proliferation by modulating SHh signaling [412]. Furthermore, the N-terminal product of SHh (ShhN) has been shown to be overexpressed in cancer and thus can be recognized as a promising target that merits further consideration [413]. Despite these encouraging findings, none of these agents has been evaluated in clinical trials, and tremendous efforts are also needed to translate related basic research into clinical practice.

#### Targeting Smo

Smo represents a primary and promising target for blocking Hh signaling for a long time. Some Smo antagonists have been tested in the clinic. Mechanically, Smo antagonists specifically bind to the transmembrane domain and the pockets in the extracellular domain of Smo so as to inhibit Hh signal transduction [344, 414].

Vismodegib (GDC‑0449) was the first FDA-approved inhibitor in 2012 that can bind to SMO to disrupt its consistent activation, primarily for the treatment of patients with recurrent, locally advanced or metastatic basal cell carcinoma (BBC) [415, 416]. Several clinical trials have been conducted to test the anticancer effects of vismodegib as monotherapy or in combination with other agents in various conditions, including metastatic pancreatic cancer, prostate cancer, gastric cancer, recurrent glioblastoma, myelofibrosis, and acute myeloid leukemia [417–421]. Most of these trials are in stages I and II, as listed in Table 2. At present, when surgery and radiotherapy are inappropriate for anticancer therapy, vismodegib remains the major option for locally advanced BCC patients [422]. Although some complete or partial antitumor responses were obtained (NCT00607724, NCT02667574, NCT01815840, NCT01367665) [423–427], disappointing results remain (NCT01088815, NCT0160118, NCT01537107) [428–430], and unexpected adverse events associated with vismodegib frequently led to interruption of treatment, ultimately leading to cancer recurrence (NCT00957229) [431]. Moreover, in the course of vismodegib treatment, researchers found a novel Smo mutation that was typically accompanied by therapeutic resistance, which limited further clinical application of Smo-based therapy [432, 433]. Hence, how to reduce treatment-associated toxic side effects and Smo mutation is the next challenge we need to overcome.

**Table 2:** Recent clinical trials targeting the Hedgehog pathway

Target	Drug name	Conditions	Combination	Phase	Current states	Trial number
SMO	Vismodegib (GDC-0449)	Basal Cell Carcinoma	-	II	Active, not recruiting	NCT02667574
		metastatic pancreatic cancer	Gemcitabine and nab-paclitaxel	II	Completed	NCT01088815
		Basal Cell Carcinoma	-	II	Completed	NCT01815840
		Advanced or Metastatic Sarcoma	RO4929097	I/II	Completed	NCT01154452
		Recurrent Childhood Medulloblastoma	-	II	Completed	NCT01239316
		Basal Cell Carcinoma	-	II	Completed	NCT01367665
		Adult Medulloblastoma	-	II	Completed	NCT00939484
		Medulloblastoma	Temozolomide	I/II	Terminated	NCT01601184
		Medulloblastoma	Chemotherapies	II	Recruiting	NCT01878617
		Locally Advanced or Metastatic Solid Tumors	-	I	Completed	NCT00607724
		Multiple Basal Cell Carcinomas	-	I	Completed	NCT02639117
		Locally Advanced Basal Cell Carcinoma, Skin Cancer	Radiation therapy	II	Completed	NCT01835626
		Basal Cell Carcinoma	Radiation therapy	II	Terminated	NCT02956889
		Breast cancer	Paclitaxel, Epirubicin, Cyclophosphamide	II	Unknown	NCT02694224
		Skin Basal Cell Carcinoma	Pembrolizumab	I/II	Completed	NCT02690948
		Basal Cell Carcinoma	-	IV	Completed	NCT02436408
		Acute Myeloid Leukemia	Ribavirin, Decitabine	II	Unknown	NCT02073838
		Intracranial Meningioma Recurrent Meningioma	FAK Inhibitor GSK2256098, Capivasertib, Abemaciclib	II	Recruiting	NCT02523014
		Basal Cell Carcinoma	-	II	Terminated	NCT02067104
		Basal Cell Carcinoma of the Skin Recurrent Skin Cancer	Mohs surgery	I	Completed	NCT01631331
		Basal Cell Carcinoma	-	II	Completed	NCT01700049
		Basal Cell Carcinoma	-	II	Terminated	NCT01898598
		Multiple Basal Cell Carcinomas	Aminolevulinic acid %20 topical solution	II	Completed	NCT01556009
		Prostate cancer	-	I	Completed	NCT02115828
		Advanced Chondrosarcomas	-	II	Active, not recruiting	NCT01267955
		Recurrent Pancreatic Carcinoma	Gemcitabine Hydrochloride	II	Completed	NCT01195415
		Metastatic Pancreatic Cancer or Solid Tumors	Erlotinib Hydrochloride, Gemcitabine Hydrochloride	I	Active, not recruiting	NCT00878163
		Advanced Stomach Cancer or Gastroesophageal Junction Cancer	Oxaliplatin, leucovorin calcium, fluorouracil	II	Completed	NCT00982592
		Prostate cancer	Goserelin Acetate, Leuprolide Acetate	I/II	Terminated	NCT01163084
		Small Cell Lung Carcinoma	Cisplatin, Cixutumumab, Etoposide	II	Completed	NCT00887159
		Keratocystic Odontogenic Tumor	-	II	Completed	NCT02366312
	Sonidegib (LDE225)	Basal Cell Carcinoma	-	II	Completed	NCT01327053
		Advanced solid tumor	-	I	Completed	NCT00880308
		Basal Cell Carcinoma	-	II	Completed	NCT01350115
		Pancreatic cancer	Gemcitabine and nab-paclitaxel	I/II	Completed	NCT02358161
		medulloblastoma	Azacitidine, Decitabine	I	Completed	NCT02129101
		lung cancer	Etoposide, Cisplatin	I	Completed	NCT01579929
		Prostate cancer	-	I	Completed	NCT02111187
		Recurrent Ovarian Cancer	-	I	Completed	NCT02195973
		Hepatocellular Carcinoma	-	I	Completed	NCT02151864
		Esophageal cancer	Everolimus	I	Completed	NCT02138929
		Multiple Myeloma	Lenalidomide	II	Completed	NCT02086552
		Basal Cell Carcinoma	Imiquimod	II	Recruiting	NCT03534947
		Pancreatic Ductal Adenocarcinoma	Gemcitabine, nab-paclitaxel	I/II	Terminated	NCT01431794
		Advanced pancreatic cancer	Fluorouracil; Leucovorin, Oxaliplatin; Irinotecan	I	Completed	NCT01485744
		Advanced solid tumor	BKM120	I	Completed	NCT01576666
		Advanced solid tumor	-	I	Completed	NCT01208831
		Solid tumors, Ovarian Cancer	Paclitaxel	I	Completed	NCT01954355
		Advanced or metastatic basal cell carcinoma	Buparlisib	II	Terminated	NCT02303041
	PF-04449913 (Glasdegib)	Acute Myeloid Leukemia	Low dose ARA-C (LDAC), decitabine, daunorubicin, Cytarabine	II	Completed	NCT01546038
		Chronic Myelomonocytic Leukemia	-	II	Completed	NCT01842646
		Hematologic Malignancies	-	II	Completed	NCT00953758
		Solid Tumors	-	I	Completed	NCT01286467
		Acute Myeloid Leukemia	Daunorubicin + cytarabine, azacitidine, placebo, Cytarabine	III	Completed	NCT03416179
		Acute Myeloid Leukemia	Gemtuzumab Ozogamicin	III	Recruiting	NCT04168502
		Acute Myeloid Leukemia	Gemtuzumab Ozogamicin	III	Terminated	NCT04093505
		Acute Myeloid Leukemia	CPX-351	II	Recruiting	NCT04231851
		Acute Myeloid Leukemia	Decitabine	II	Terminated	NCT04051996
		Hematologic Malignancies	Azacitidine	I	Completed	NCT02367456
		Acute Myeloid Leukemia	Avelumab,azacitidine, gemtuzumab ozogamicin, Glasdegib, glasdegib maleate, venetoclax	I/II	Active, not recruiting	NCT03390296
	LY2940680 (Taladegib)	Localized Esophageal or Gastroesophageal Junction Cancer	Carboplatin, paclitaxel and radiation therapy	I/II	Completed	NCT02530437
		Small Cell Lung Carcinoma	Carboplatin, etoposide, placebo	I/II	Terminated	NCT01722292
		Neoplasm Metastasis	-	I	Completed	NCT01919398
		Advanced Cancer	-	I	Completed	NCT01226485
		Advanced or Metastatic Solid Tumors	Taladegib, abemaciclib, cisplatin, gemcitabine, Carboplatin, LY3023414	I	Completed	NCT02784795
	TAK-441	Advanced nonhematologic malignancies, Basal Cell Carcinoma	-	I	Completed	NCT01204073
	BMS-833923	Advanced or metastatic cancer	-	I	Completed	NCT00670189
		small cell lung cancer	Carboplatin, etoposide	I	Completed	NCT00927875
		Metastatic gastric, gastroesophageal, or esophageal adenocarcinomas	Cisplatin, capecitabine	I	Completed	NCT00909402
		Leukemia	Dasatinib	I/II	Completed	NCT01218477
	LEQ506	Advanced solid tumors	-	I	Completed	NCT01106508
	IPI-926	Advanced Pancreatic Adenocarcinoma	FOLFIRINOX	I	Completed	NCT01383538
		Recurrent head and neck cancer	Cetuximab	I	Completed	NCT01255800
		Advanced chondrosarcoma	-	II	Completed	NCT01310816
		Metastatic pancreatic cancer	Gemcitabine	I/II	Completed	NCT01130142
		Metastatic solid tumor	-	I	Completed	NCT00761696
GLI	arsenic trioxide	Basal Cell Carcinoma	-	I/II	Completed	NCT01791894
		Advanced Neuroblastoma or Other Childhood Solid Tumors	-	II	Completed	NCT00024258
		High-Grade Glioma	Temozolomide and radiation therapy	I	Completed	NCT00720564
		Newly Diagnosed Gliomas	Radiation therapy	I	Completed	NCT00095771
		Recurrent Malignant Glioma	-	I	Completed	NCT00185861
		Acute Promyelocytic Leukemia	Tretinoin	II	Completed	NCT01404949

The relevant clinical trial data were obtained from the registration on ClinicalTrials.gov

Sonidegib, also known as LDE‑225, is a synthetically derived, new molecular entity that exerts its function by binding and inhibiting Smo receptors and is the second Smo inhibitor approved by the FDA in 2015 for the treatment of patients with locally advanced BCC not amenable to curative surgery or radiation therapy [434–436]. The use of sonidegib was typically well tolerated and yielded impressive clinical efficacy with reported objective responses, and there was a significant association between the anticancer efficiency and the Hh signaling activation monitored by gene expression (NCT01350115, NCT01327053, NCT00880308) [437–442]. Consistently, in a recent observational, retrospective, single-center study, researchers evaluated the clinical efficacy and safety of sonidegib in patients with BCC. The results showed that all enrolled subjects benefited from the treatment, either with a response or stabilization of the disease, supporting the further application of sonidegib in the clinic [443]. Nevertheless, in patients with BCC who were resistant to vismodegib, the same therapeutic resistance was also observed, thereby hampering sonidegib’s anticancer effect (NCT01529450) [444]. Future efforts in targeting Smo in cancer should focus on approaches addressing and overcoming these resistance mechanisms [42, 445]. In addition to BCC, previous and ongoing phased clinical trials have also been conducted to test the activity and response of sonidegib in other cancer types, including hepatocellular carcinoma, lung cancer, pancreatic adenocarcinoma, prostate cancer and esophageal cancer (Table 2).

PF-04449913 (Glasdegib) is another Smo inhibitor developed by Pfizer and has been approved by the FDA for treating both solid tumors and hematological malignancies [446–449]. Previous phased clinical trials identified its safety, tolerance, and potential antitumor efficacy in myeloid malignancies, glioblastoma and other advanced solid tumors [450–452]. Puhlished data support its further evaluation and motivate the subsequent initiation of phase III clinical trials as monotherapy or in combination with standard chemotherapy, which is summarized in Table 2.

Taladegib (LY-2940680) can bind to the Smo receptor, thereby blocking the propagation of Hh signaling. Currently, this synthetic small molecule inhibitor is being tested in several clinical trials to treat patients with advanced solid tumors [453]. TAK-441 is a highly potent and oral SMO inhibitor that has activity against both Hh ligand overexpression and mutation-driven Hh signaling pathway activation [454].

Furthermore, other Smo inhibitors, including LDE225, LEQ506, BMS-833923, IPI-926, LY2940680 and GDC0499, are currently under clinical investigation [455].

#### Targeting Gli

As previously mentioned, Gli represents a crucial transcription factor that can act as a downstream effector of canonical and noncanonical Hh signaling. Consequently, targeting Gli has the potential to overcome therapeutic resistance caused by Smo inhibitors [456, 457]. Simultaneously, extraordinary efforts have been made to discover and design reagents targeting Gli to achieve Hh pathway inhibition.

Initially, identified from cellular screening assays in 2007, GANT58 and GANT61 were demonstrated to be selective inhibitors of Hh-driven tumor growth because of their capacity to downregulate the transcriptional activity of Gli1 and Gli2 [458–461]. Since then, a substantial number of studies have explored their antitumor effects and have shown that GANT61 can inhibit malignant behavior, induce autophagy and apoptosis, and enhance the therapeutic sensitivity of tumor cells both in vitro and in vivo [462–466]. However, no clinical trials using GANT61 for treating human cancer are currently ongoing.

Arsenic trioxide (ATO) is another Gli inhibitor with the potential to decrease Gli transcriptional activity by binding to Gli1 and Gli2 and has been reported to delay tumor progression in preclinical studies [467, 468]. Clinically, ATO is evaluated for the treatment of different cancer types, including lung cancer, acute myeloid leukemia, and hepatocellular carcinoma [469–471]. (Table 2) Pirfenidone, an antifibrotic agent, selectively destabilizes Gli2 [472]. Imiquimod, an agonist of toll-like receptors 7 and 8, directly suppresses Hh signaling by stimulating PKA-mediated Gli phosphorylation [473].

## Conclusion and future perspective

Since the first discovery of conserved key signaling pathways, such as Notch and Hh, in Drosophila, researchers have been persistently exploring their structure, regulatory mechanisms, physiology, and pathology and developing therapeutic strategies to target some of the signaling pathway components. This review summarizes the work that has been done in both the Notch and Hh signaling pathways and clarifies the scope for further basic research and the value of developing clinical applications for Notch and Hh signaling in cancer, where the activation of Hh is usually associated with tumor development. Inhibitors of its components (SHh, Smo, Gli) have been quite effective in some tumors (BCC) and some unsatisfactory. One of the most interesting aspects of NOTCH signaling is its context-dependent nature, which means that it possesses pro- or anticancer effects under different conditions. First, different types of ligands in the pathway have their own roles or different combinations of ligands and receptors, producing distinct effects. In addition, Notch function differs in diverse tumor types or even different subclones of the same tumor, which is perhaps related to Notch-dependent proximity secretion. Of course, the downstream gene expression of Notch contains both positive and negative regulators of tumor growth, perhaps also related to its double-edged sword effect.

NOTCH- and Hh-targeted therapies have been studied for decades but have failed to meet expectations. The main reasons for this include high pan-inhibition-induced organismal toxicity, low affinity for drug delivery systems, upregulation of bypass activation pathways, and the mechanism-driven group of individual drugs whose activity can easily be overestimated in preclinical models. The development of new drugs targeting molecules with higher specificity within the pathway and affinity antibody‒drug couples may be a countermeasure. New immunotherapeutic strategies, including DC pulse vaccines, CAR-T cells and DLL3-CAR-NK-cell therapies, are more attractive. Combination therapies with radiotherapy, chemotherapy, immunotherapy and other small molecule inhibitors can be considered. miRNAs interfere with both the Notch and Hh pathways and can be used as potential biomarkers and therapeutic approaches, although their delivery still poses technical challenges. As intradermal lysoviral therapy has entered the standard of care phase of oncology, viruses could be considered designed to respond to high Notch and Hh activity within the target cells. The antitumor effects of natural products of related pathways should not be underestimated [474, 475].

With the unveiling of findings on the mechanisms of embryonic development, a complex circuit formed between the two signaling pathways has been laid out. Notch and Hh signaling control each other in this feedback fashion. A tightly controlled loop prevents uncontrolled proliferation and incorrect patterns. It is reasonable to speculate whether there is a break in the control loop in tumor cell populations that proliferate out of control and whether the re-establishment of feedback can in turn gently promote tumor regression, all of which deserve further exploration. The field of cell signaling crosstalk is rapidly expanding, and one would expect them all to be part of the same conversation, taking a more coordinated and comprehensive view of the problem and solving it.

## Data Availability

Not applicable.
